# Process Optimization and Uncertainty Analysis of IoT‐Integrated Cyclic Microwave‐Assisted Extraction (CMAE) for Vanillin Recovery

**DOI:** 10.1155/ijfo/4384246

**Published:** 2026-09-28

**Authors:** Narongrit Tipcompor, Thanapon Saengsuwan, Narathip Sujinda, Sujitra Funsueb, Watcharaporn Thaharn

**Affiliations:** ^1^ Chemistry Program, Faculty of Education, Chiang Rai Rajabhat University, Chiang Rai, Thailand; ^2^ Energy Engineering and Electric Technology Program, Faculty of Industrial Technology, Chiang Rai Rajabhat University, Chiang Rai, Thailand; ^3^ Faculty of Agriculture at Kamphaeng Saen, Kasetsart University, Kamphaeng Saen Campus, Nakhon Pathom, Thailand, ku.ac.th; ^4^ Department of Chemistry, Faculty of Science, Chiang Mai University, Chiang Mai, Thailand, cmu.ac.th

**Keywords:** cyclic microwave-assisted extraction, real-time temperature monitoring, response surface methodology, *Vanilla planifolia*, vanillin

## Abstract

Vanillin, the principal flavor compound in vanilla, is a high‐value natural product whose conventional extraction is often limited by low efficiency and high energy consumption. Although microwave‐assisted extraction (MAE) has improved extraction efficiency, most systems rely on continuous operation with limited real‐time process monitoring. To address this limitation, an IoT‐integrated cyclic microwave‐assisted extraction (CMAE) system was developed for vanillin recovery from *Vanilla planifolia*. The system incorporated an infrared temperature sensor, a solid‐state relay, an ESP32 microcontroller, and a Node‐RED interface to enable real‐time temperature monitoring and programmable cyclic microwave operation. Response surface methodology identified optimal conditions of 436 W microwave power, 16.93 min extraction time, 3.45 g sample mass on a dry‐weight basis, and 75% ethanol, with a predicted vanillin content of 3.13% DW, equivalent to a yield of 31.29 mg/g DW. Monte Carlo simulation yielded a 95% uncertainty interval of 29.41–33.16 mg/g DW (2.94%–3.32% DW), whereas sensitivity analysis identified microwave power and ethanol concentration as the most influential variables. Experimental validation under approximately energy‐equivalent conditions of 380 W for 19.44 min and 500 W for 14.78 min yielded 30.43 and 30.98 mg/g DW, respectively, with deviations from the predicted value below 3%. Assuming equivalent vanillin recovery, cyclic operation reduced the calculated nominal microwave energy input and specific energy consumption (SEC) by 16.7% relative to a calculated continuous‐operation baseline. The developed CMAE system provides a data‐driven extraction platform integrating IoT‐based monitoring, programmable cyclic operation, process optimization, and uncertainty analysis, with potential application in energy‐conscious phytochemical extraction.

## 1. Introduction

Vanillin (4‐hydroxy‐3‐methoxybenzaldehyde), recognized as the primary flavor constituent of vanilla, is among the most valuable and widely utilized flavoring compounds globally [1], with applications spanning the food [2], fragrance [3], pharmaceutical [4], and cosmetic industries [5]. The compound is naturally derived from the cured pods of *Vanilla planifolia* and commands a premium market value, reinforced by the expanding demand for naturally sourced vanillin relative to synthetic alternatives [6]. To enhance the recovery of vanillin from plant matrices, a variety of extraction techniques have been explored, including traditional liquid–liquid and liquid–solid extraction [7], as well as more advanced approaches such as supercritical fluid extraction (SFE) [8], solid phase microextraction (SPME), and ultrasound‐assisted extraction (UAE) [9]. However, conventional solvent‐based and distillation‐derived processes often require long processing times, substantial energy input, and large solvent volumes, limiting their economic and environmental sustainability [10].

The advent of microwave‐assisted extraction (MAE) has marked a paradigm shift in phytochemical recovery, offering a compelling green alternative to traditional methods. Through microwave–matter interactions (primarily ionic conduction and dipole rotation), MAE enables rapid and volumetric heating within the solvent–plant matrix, enhancing cell disruption, improving mass transfer, and increasing extraction efficiency while markedly reducing processing time [11–14]. Several studies have demonstrated that MAE can significantly enhance vanillin yield and preserve antioxidant properties in extracts obtained from *Vanilla planifolia* [15–18].

Despite these advantages, the reproducibility and energy management of MAE remain challenging. Continuous microwave irradiation can promote nonuniform heating and localized thermal stress, potentially accelerating the degradation of thermolabile compounds under severe processing conditions [14, 19]. In addition, limited real‐time monitoring and reliance on fixed operating protocols may restrict process consistency and complicate future scale‐up [20, 21]. Previous vanillin‐extraction studies have largely focused on optimizing fixed or continuous microwave conditions, whereas the combined application of programmable cyclic irradiation, IoT‐based temperature monitoring, statistical optimization, and uncertainty analysis remains limited [18]. To address this gap, the present study developed an IoT‐integrated cyclic microwave‐assisted extraction (CMAE) system incorporating real‐time temperature monitoring and programmable microwave ON/OFF operation. Cyclic irradiation provides periodic OFF intervals that may support thermal equilibration and reduce unnecessary microwave exposure [22]. The IoT platform enables continuous monitoring, automated data recording, and supervisory operation through an ESP32–Node‐RED interface, consistent with smart‐processing concepts associated with Industry 4.0 [23]. The novelty of this work lies in integrating programmable cyclic microwave operation, IoT‐based real‐time temperature monitoring, response surface methodology (RSM), Monte Carlo uncertainty and sensitivity analyses, and experimental validation within a single framework for vanillin recovery. This approach provides a data‐driven basis for evaluating process conditions, prediction uncertainty, and the potential energy advantage of cyclic microwave operation [23, 24].

Accordingly, this study had three objectives. First, an IoT‐integrated CMAE system was developed for vanillin extraction with real‐time temperature monitoring and programmable cyclic operation. Second, RSM was applied to model and optimize microwave power, extraction time, sample mass, and ethanol concentration, whereas Monte Carlo simulation was used to quantify prediction uncertainty and identify influential process variables [25, 26]. Third, the optimized operating region was validated using approximately energy‐equivalent power–time combinations, and the calculated nominal microwave energy input and specific energy consumption (SEC) were compared with a calculated continuous‐operation baseline under an equal‐recovery assumption. Overall, the study is aimed at establishing a statistically supported and energy‐conscious extraction framework for high‐value phytochemical recovery.

## 2. Materials and Methods

### 2.1. Plant Material and Chemical Reagents

Cured *Vanilla planifolia* beans were obtained from Siam Vanilla Akha (Doi Tung, Chiang Rai, Thailand), ground using a laboratory mill (A11 basic, IKA, Germany), and passed through a 0.630 mm sieve to obtain a uniform particle size suitable for efficient solvent contact and vanillin mass transfer during extraction. This particle size was selected following the methodology reported by Dong et al. [11]. The powdered pods exhibited an initial moisture content of 28.5*%* ± 1.2*%*, determined by oven drying according to AOAC Method 925.10, and this value was used to calculate the as‐received sample mass corresponding to 2–6 g dry weight (DW) and to express vanillin yield on a DW basis. The samples were vacuum‐sealed in low‐density polyethylene (LDPE) bags and stored at 4°C until further analysis. HPLC‐grade methanol and ultrapure water were used as chromatographic mobile phases, whereas analytical‐grade ethanol (≥ 99.5%) was used as the extraction solvent (RCI Labscan, Bangkok, Thailand). Vanillin standard (≥ 99% purity) and Folin–Ciocalteu reagent were purchased from Sigma‐Aldrich (St. Louis, Missouri, United States).

### 2.2. CMAE System Design and Instrumentation

The CMAE system (Figure 1) was developed by modifying a commercial microwave oven (Samsung ME711K, 700 W, 2.45 GHz) to enable real‐time temperature monitoring and programmable cyclic microwave operation for extraction applications [27]. The primary modifications included the integration of a thermal monitoring unit and a programmable control unit for executing predefined microwave ON/OFF cycles.

**Figure 1:**
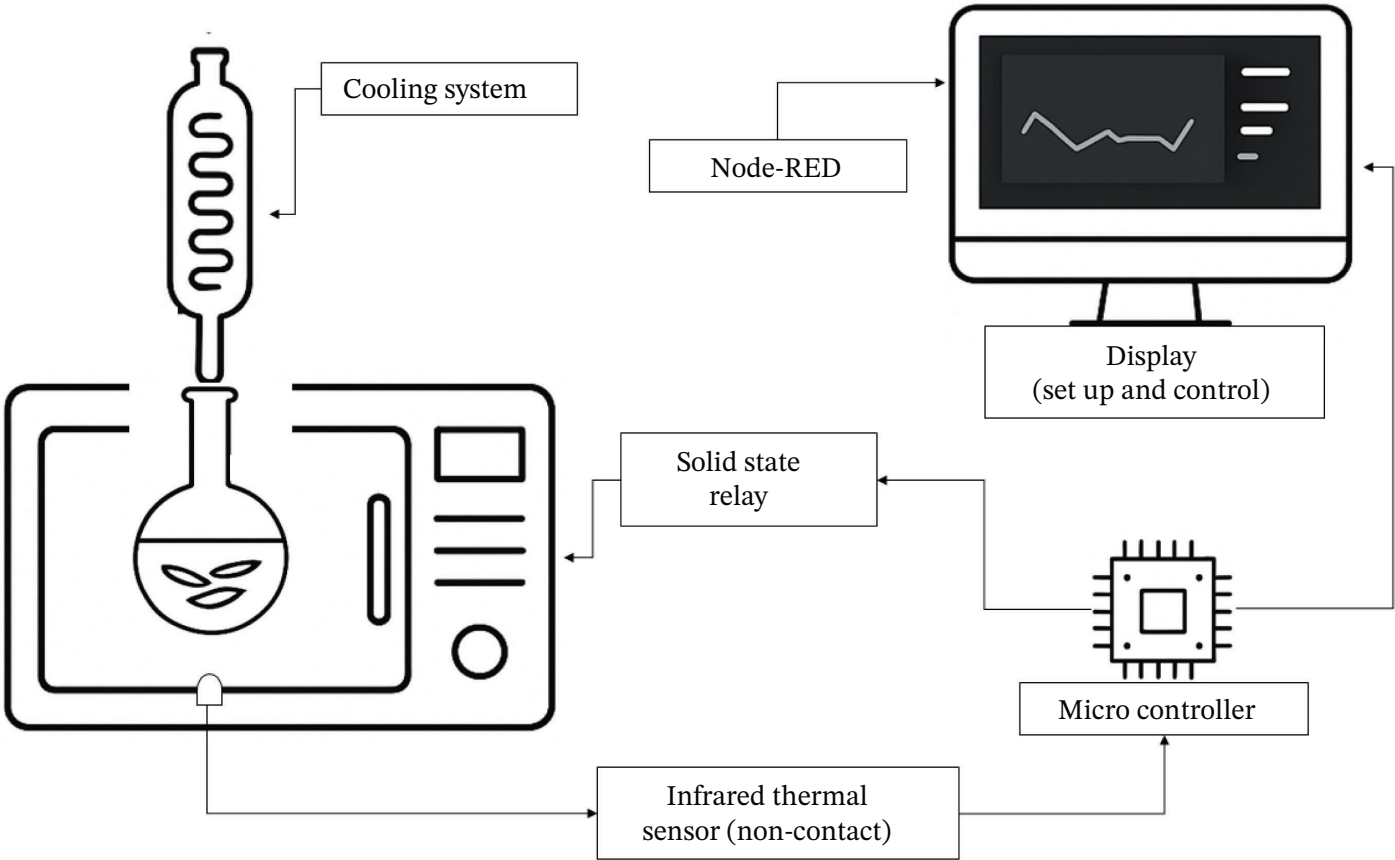
Schematic diagram of the IoT‐integrated CMAE system with real‐time temperature monitoring and programmable cyclic microwave control.

The thermal monitoring subsystem comprised a noncontact infrared temperature sensor (MLX90614ESF‐BAA) with an I^2^C digital interface and an accuracy of ±0.5°C over the temperature range of 0°C–50°C. The sensor was mounted on a polytetrafluoroethylene (PTFE) bracket inside the microwave cavity and positioned 5 cm from the extraction vessel to enable continuous monitoring of the vessel surface temperature while minimizing interference with microwave exposure [28].

Microwave switching was controlled using a solid‐state relay (SSR‐40DA, Fotek), which enabled rapid execution of the programmed ON/OFF sequence. An ESP32‐WROOM‐32D microcontroller served as the central processing unit and executed the control program developed using the Arduino integrated development environment (IDE, Version 2.0.4).

The control program applied microwave irradiation for 25 s followed by a 5 s OFF period [29], corresponding to a duty factor of 25/30, or 0.833. This predefined duty cycle was maintained throughout all experimental runs to provide consistent and reproducible microwave exposure. During the OFF periods, microwave irradiation was interrupted, whereas temperature monitoring continued. A Node‐RED dashboard (Version 3.0.2), hosted on a local server, provided a graphical user interface for real‐time monitoring of temperature, microwave operating status, power setting, and cycle count, as well as supervisory control of system operation. Microwave leakage was verified using a Narda Safety Test Solutions HI‐1501 leakage detector and was maintained below 5 mW/cm^2^.

### 2.3. Extraction Procedure and Experimental Design

Ground cured *Vanilla planifolia* beans (2–6 g on a DW basis) were used for the extraction experiments. Based on the initial moisture content (28.5% ± 1.2%), approximately 2.80–8.39 g of the as‐received sample was weighed for each experiment. The samples were mixed with 100 mL of aqueous ethanol in a 250‐mL round‐bottom flask, corresponding to solid‐to‐solvent ratios ranging from 1:50 to 1:16.7 g/mL on a DW basis. The mixtures were then subjected to MAE using a system equipped with a reflux condenser. The four independent variables investigated in the central composite design (CCD) were microwave power (A), extraction time (B), sample weight (C), and ethanol concentration (D), with their coded and actual levels presented in Table 1. The experimental design comprised 31 runs, including factorial points, axial points, and replicated center points, allowing estimation of quadratic effects and pure error. Immediately after irradiation, the extracts were filtered through Whatman No. 1 filter paper to remove solid residues. The filtrates were quantitatively transferred into 100‐mL volumetric flasks and made up to volume with the extraction solvent. A 100 *μ*L aliquot was subsequently diluted to 10 mL with ethanol prior to HPLC analysis. All experiments were performed in triplicate, and the run order was randomized to minimize systematic error and ensure statistical reliability.

**Table 1:** Independent variables and their levels in the central composite design (CCD).

Variable	Symbol	−2	−1	0	+1	+2
Microwave power (W)	A	120	250	380	500	700
Time (min)	B	5	10	15	20	25
Sample weight (g, DW)	C	2	3	4	5	6
Ethanol concentration (%)	D	60	67.5	75	82.5	90

### 2.4. Analytical Methods and Validation

#### 2.4.1. Vanillin Quantification by High‐Performance Liquid Chromatography

Vanillin content was quantified using a PerkinElmer LC 300 HPLC system (PerkinElmer Inc., Waltham, Massachusetts, United States) equipped with a Brownlee SPP C18 column (4.6 × 100 mm, 2.7 *μ*m) and a photodiode array (PDA) detector. Isocratic separation was performed using a mobile phase of methanol:acidified water (15:85, v/v), where the aqueous component was prepared by adding 0.1% (v/v) acetic acid to ultrapure water [11]. The flow rate was 0.5 mL/min, the column temperature was maintained at 30°C, the injection volume was 1 *μ*L, and the detection wavelength was set at 254 nm. Samples were filtered through 0.45 *μ*m membrane filters prior to analysis. Method validation was conducted following the International Council for Harmonisation Q2 (R1) guidelines. Calibration curves were constructed using authentic vanillin standards over a concentration range of 25−200 *μ*g/mL and demonstrated excellent linearity with a coefficient of determination (*R* ^2^) of 0.99995 (Supporting Information 1: Figure S1).

#### 2.4.2. Total Phenolic Content (TPC) Determination

TPC was determined using the Folin–Ciocalteu method [13]. Briefly, 0.5 mL of appropriately diluted extract was mixed with 2.5 mL of 10% w/v Folin–Ciocalteu reagent. After a 5‐min incubation, 2.0 mL of 7.5% w/v Na_2_CO_3_ solution was added, and the mixture was incubated in the dark for 60 min. Absorbance was measured at 765 nm using a UV‐visible spectrophotometer. TPC was expressed as milligrams of gallic acid equivalents per gram of DW based on a calibration curve (*R* ^2^ = 0.9993).

### 2.5. RSM and Statistical Modeling

A second‐order polynomial model was fitted to the experimental data obtained from the CCD (Supporting Information 2: Table S1) using Design‐Expert software (Version 13, Stat‐Ease Inc., Minneapolis, Minnesota, United States) [25]. The general form of the model was as follows:

(1) Y=β0+∑i=1kβiXi+∑i=1kβiiXi2+∑i=1k−1∑j=i+1kβijXiXj+ε,

where *Y* is the predicted response expressed as vanillin content (% DW), which was converted to milligrams per gram of DW by multiplying the percentage value by 10; *β*_0_ is the constant coefficient; *β*_*i*_, *β*_*i* *i*_, and *β*_*i* *j*_ are the linear, quadratic, and interaction coefficients, respectively; *X*_*i*_ and *X*_*j*_ represent the coded values; *k* is the number of independent variables; and *ε* represents the random error term. The statistical significance of the overall model and individual model terms was evaluated by analysis of variance (ANOVA) at a significance level of *p* < 0.05. The full quadratic model was retained to preserve model hierarchy and was used for response surface analysis, numerical optimization, uncertainty analysis, and prediction. Although several individual interaction terms were not statistically significant, they were retained as components of the hierarchical full quadratic model. Model adequacy was assessed using *R* ^2^, adjusted *R* ^2^, predicted *R* ^2^, adequate precision, and the lack‐of‐fit test [14, 25]. The assumptions and reliability of the fitted model were further evaluated using residual and influence diagnostics, including the normal probability plot of internally studentized residuals, the residuals‐versus‐predicted plot, leverage, Cook′s distance, and DFFITS.

### 2.6. Monte Carlo Simulation for Uncertainty and Sensitivity Analysis

To quantify the uncertainty associated with the model predictions under the optimized extraction conditions, a Monte Carlo simulation was performed with 10,000 iterations using Python 3.6 [30]. The framework accounted for three uncertainty sources: (1) model parameter uncertainty derived from the variance–covariance matrix of the regression coefficients, (2) measurement uncertainty represented by an HPLC relative standard deviation (RSD) of 1.5%, and (3) operational variability in microwave power, extraction time, sample mass, and ethanol concentration [31].

The regression coefficients were sampled jointly from a multivariate normal distribution, using the estimated coefficients of the full quadratic model as the mean vector and their variance–covariance matrix as the covariance matrix. This procedure preserved the correlations among the estimated model coefficients. Microwave power and extraction time were sampled from uniform distributions within their operational precision limits of ±5 W and ±0.1 min, respectively. Sample mass and ethanol concentration were also sampled from uniform distributions using their experimentally established preparation tolerances of ±0.01 g and ±0.5% (v/v), respectively. Analytical error was modeled as a multiplicative normal error with a mean of zero and an RSD of 1.5%.

For each iteration, the sampled regression coefficients and process variables were substituted into the full quadratic model to calculate the predicted vanillin content (% DW), which was subsequently converted to vanillin yield (milligrams per gram of DW) by multiplying the percentage value by 10. The analytical error was subsequently incorporated into the simulated prediction. The mean, 2.5th percentile, and 97.5th percentile of the simulated output distribution were used to determine the mean predicted yield and its Monte Carlo–derived 95% uncertainty interval. To estimate uncertainty in the optimized process variable values reported in Table 2, numerical optimization was repeated for each sampled full‐model realization. The distributions of the resulting optimal microwave power, extraction time, sample mass, ethanol concentration, and predicted yield were summarized using their 2.5th and 97.5th percentiles. The overall uncertainty propagation procedure is illustrated in Figure 2. To identify the relative contribution of each input variable to prediction uncertainty, a sensitivity analysis was performed using the standardized regression coefficient (SRC) method. A multiple linear regression model was fitted between the sampled process variables and the corresponding simulated outputs. The SRC for each input variable (*X*_*i*_) was calculated as follows:

(2) SRCi=biσXiσY,

where *b*_*i*_ is the regression coefficient obtained from the linear regression of the simulated output on the sampled input variables and $\sigma_{X_{i}}$ and *σ*_*Y*_ are the standard deviations of the corresponding input variable and simulated output, respectively. Variables with |SRC| ≥ 0.1 were considered influential. The relative contribution of each process variable to output variance was estimated from the sensitivity analysis and is reported in Table 3.

**Table 2:** Optimized CMAE conditions, Monte Carlo–derived 95% uncertainty intervals, and practical operating settings.

Parameter	Optimal point	Monte Carlo–derived 95% uncertainty interval	Practical setting
Power (W)	436	421.5–451.2	436
Time (min)	16.93	16.1–17.8	17.0
Mass (g, DW)	3.45	3.31–3.59	3.45
Ethanol (%)	75.0	72.4–77.6	75.0
Predicted yield (mg/g DW)	31.29	29.41–33.16	—

**Figure 2:**
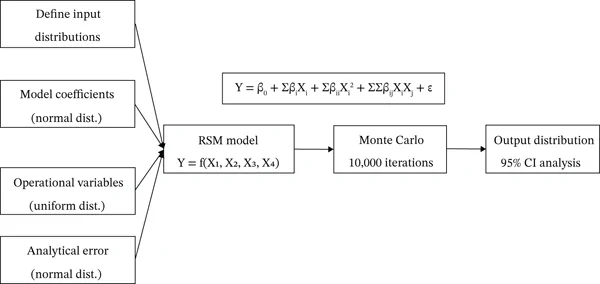
Flowchart of the Monte Carlo simulation procedure for uncertainty propagation and sensitivity analysis.

**Table 3:** Sensitivity analysis of input variables affecting the predicted vanillin yield obtained from Monte Carlo simulation.

Parameter	Absolute standardized regression coefficient (|*S* *R* *C*|)	Relative importance based on normalized *S* *R* *C* ^2^ (%)
Microwave power (*X*_1_)	0.45	50.0
Ethanol concentration (*X*_4_)	0.38	35.6
Extraction time (*X*_2_)	0.22	11.9
Sample mass (*X*_3_)	0.10	2.5
Total	—	100.0

### 2.7. Energy Consumption Analysis

Energy consumption of the CMAE system was characterized using the calculated SEC, defined as the nominal microwave energy input per unit mass of vanillin recovered:

(3) SEC=PTon1000mvanillinkJg,

where *P* is the applied microwave power (watts), *T*_on_ is the cumulative ON‐time during cyclic operation (seconds), and *m*_vanillin_ is the mass of vanillin recovered per extraction batch (grams). The factor of 1000 converts the calculated energy from joules to kilojoules. Because energy was calculated from the applied microwave power rather than measured using a wall‐plug power meter, the resulting values represent nominal microwave energy input rather than the total electrical energy consumed by the complete system. For cyclic operation with 25 s of microwave irradiation followed by a 5‐s OFF period, the cumulative ON‐time was calculated as follows:

(4) Ton=ttotaltcycle,ONtcycle,ON+tcycle,OFF=ttotal2530,

where *t*_total_ is the total extraction time. A calculated continuous‐operation baseline was established using the same applied microwave power and total extraction time but with uninterrupted microwave irradiation. The nominal microwave energy input for continuous operation was calculated as follows:

(5) Econtinuous=Pttotal1000 kJ,

where *t*_total_ is expressed in seconds. The nominal microwave energy input for cyclic operation was similarly calculated as *E*_cyclic_ = *P* *T*_on_/1000. The percentage reduction in nominal microwave energy input relative to continuous operation was calculated as follows:

(6) Energy reduction %=Econtinuous−EcyclicEcontinuous×100.

For the SEC comparison, equivalent vanillin recovery was assumed for cyclic and continuous operation. Therefore, the calculated SEC reduction reflects the difference in nominal microwave irradiation time and should not be interpreted as an experimentally measured comparison of extraction efficiency between the two operating modes.

### 2.8. Statistical Analysis

All experiments were conducted in triplicate, and the results are presented as mean ± standard deviation. The full quadratic response surface model and the significance of the overall model and individual model terms were evaluated by ANOVA using Design‐Expert Version 13 (Stat‐Ease Inc., Minneapolis, Minnesota, United States). Statistical significance was defined at *p* < 0.05. Monte Carlo simulation and sensitivity analysis were performed using Python 3.6, as described in Section 2.6.

## 3. Results and Discussion

### 3.1. Model Fitting and Statistical Analysis

The experimental data for vanillin content, expressed as % DW, obtained from the CCD, were fitted to a full second‐order polynomial model [14, 25]. The ANOVA for the full quadratic model is presented in Table 4. The full model was retained to preserve model hierarchy, including the two‐factor interaction terms, and was used for response surface analysis and numerical optimization.

**Table 4:** Analysis of variance (ANOVA) for the full quadratic model of vanillin content.

Source	Sum of squares	df	Mean square	*F*‐value	*p* value	Significance
Model	4.3100	14	0.3079	8.80	< 0.0001	Significant
*X*_1_: Power	0.2660	1	0.2660	7.61	0.0140	^∗^
*X*_2_: Time	0.2500	1	0.2500	7.15	0.0166	^∗^
*X*_3_: Mass	0.1740	1	0.1740	4.98	0.0401	^∗^
*X*_4_: Solvent	0.0740	1	0.0740	2.12	0.1648	—
${X_{1}}^{2}$	1.0800	1	1.0800	30.87	< 0.0001	^∗^
${X_{2}}^{2}$	0.8530	1	0.8530	24.39	0.0001	^∗^
${X_{3}}^{2}$	0.4450	1	0.4450	12.73	0.0023	^∗^
${X_{4}}^{2}$	1.4600	1	1.4600	41.75	< 0.0001	^∗^
*X*_1_ *X*_2_	0.0070	1	0.0070	0.20	0.6670	—
*X*_1_ *X*_3_	0.0880	1	0.0880	2.53	0.1328	—
*X*_1_ *X*_4_	0.0420	1	0.0420	1.20	0.2888	—
*X*_2_ *X*_3_	0.0000	1	0.0000	0.00	0.9402	—
*X*_2_ *X*_4_	0.0150	1	0.0150	0.43	0.5176	—
*X*_3_ *X*_4_	0.0540	1	0.0540	1.55	0.2325	—
Residual	0.5590	16	0.0349	—	—	—
Lack of fit	0.4769	10	0.0477	3.49	0.0699	Not significant
Pure error	0.0821	6	0.0137	—	—	—
Cor total	4.8690	30	—	—	—	—
*R* ^2^	0.8851					
Adjusted *R* ^2^	0.7846					
Predicted *R* ^2^	0.6188					

∗ (*p* < 0.05).

The model exhibited an *F*‐value of 8.80 with a probability value of *p* <0.0001, indicating that the model was highly significant. The lack‐of‐fit *F*‐value was 3.49 (*p* = 0.0699), which was not statistically significant at the 5% level, indicating that no significant lack of fit was detected within the investigated design space. However, because the lack‐of‐fit *p* value was relatively close to 0.05, the adequacy of the model was further assessed using residual and influence diagnostics, as described in Section 3.1.1.

The full quadratic model exhibited an *R* ^2^ of 0.8851, indicating that 88.51% of the total variability in vanillin content was explained by the fitted model. The adjusted *R* ^2^ and predicted *R* ^2^ values were 0.7846 and 0.6188, respectively. The difference between these values was 0.1658, which was below 0.20, indicating reasonable agreement between the explanatory and predictive performance of the model within the investigated design space. Experimental validation was subsequently performed to further evaluate the predictive adequacy of the model. Therefore, the predictive performance of the model was further evaluated through residual diagnostics and experimental validation rather than being inferred from the goodness‐of‐fit statistics alone. The full quadratic polynomial model for vanillin content (*Y*, % DW) in terms of coded factors was as follows:

(7) Y=2.990.16420.42780.1347+X1−X2−X3+0.08780.04100.07400.0512X4+X1X2+X1X3−X1X4+0.00710.06180.05800.1463X2X3+X2X4+X3X4−X12−0.69480.12540.2273X22−X32−X42,

where *X*_1_, *X*_2_, *X*_3_, and *X*_4_ represent the coded values of microwave power, extraction time, sample mass, and ethanol concentration, respectively. Vanillin content expressed as % DW was converted to vanillin yield in milligrams per gram of DW by multiplying the percentage value by 10.

The positive linear coefficient of microwave power (*X*_1_) indicates that increasing microwave power from the center point initially increased vanillin recovery, whereas the negative linear coefficients of extraction time (*X*_2_) and sample mass (*X*_3_) indicate decreasing tendencies as these factors increased around the center of the experimental region. The linear effects of microwave power, extraction time, and sample mass were statistically significant (*p* < 0.05), whereas the positive linear effect of ethanol concentration (*X*_4_) was not statistically significant (*p* = 0.1648). These linear coefficients should be interpreted together with their corresponding quadratic and interaction terms rather than as simple monotonic effects throughout the entire factor ranges.

The negative linear coefficient of sample mass indicates that increasing the sample mass around and beyond the optimal region may reduce vanillin recovery. This effect may be attributed to nonuniform microwave energy distribution, reduced solvent penetration into the plant matrix, and increased mass transfer resistance at higher solid loadings [32].

The quadratic terms for microwave power, extraction time, sample mass, and ethanol concentration were all statistically significant and had negative coefficients. These negative quadratic coefficients indicate downward curvature of the response surfaces and the presence of intermediate operating conditions that maximize vanillin recovery within the investigated ranges. This behavior is consistent with the combined effects of microwave energy transfer, solvent properties, mass transfer resistance, and possible thermal degradation under excessive process conditions [33].

None of the two‐factor interaction terms was statistically significant within the investigated ranges. Nevertheless, the interaction terms were retained to preserve the hierarchy of the full quadratic model. Their individual effects should therefore be interpreted cautiously. The absence of statistically significant interaction terms indicates that no interactions were statistically detected within the investigated design space, but it should not be interpreted as evidence that the process variables were physically independent.

#### 3.1.1. Model Validation Through Residual Diagnostics

The adequacy of the full quadratic model was further evaluated using residual and influence diagnostics. The normal probability plot of externally studentized residuals (Supporting Information 3: Figure S2a) showed that the residuals approximately followed the diagonal reference line, supporting the assumption of approximate normality. The internally studentized residuals ranged from −2.140 to 2.358, whereas the externally studentized residuals ranged from −2.452 to 2.827. No residual exceeded the commonly used screening limit of ±3, indicating that no observation was identified as an extreme residual outlier.

The residuals‐versus‐predicted plot (Supporting Information 3: Figure S2b) showed that the residuals were distributed around zero without a clear systematic trend, curvature, or funnel‐shaped pattern. This distribution supported the assumptions of approximately constant variance and the absence of an evident systematic model bias.

For the full quadratic model, which contained 15 estimated parameters including the intercept (*p* = 15) and 31 observations (*n* = 31), the average leverage was *p*/*n* = 0.484, and twice the average leverage was approximately 0.968. None of the observations exceeded this leverage screening threshold. Run 18 exhibited the highest leverage value of 0.779, but this value remained below the screening threshold (Supporting Information 4: Table S2).

Using the DFFITS screening criterion applied to the diagnostic output, $2\sqrt{p/\left(n-p\right)}$, the threshold was approximately ±1.936. Runs 4, 13, 18, 20, and 23 exceeded this threshold, with DFFITS values of 2.625, −2.819, 2.629, 2.233, and 3.302, respectively. Among these observations, Run 23 exhibited the largest absolute DFFITS value and the highest Cook′s distance of 0.506. Nevertheless, all Cook′s distance values remained below the commonly used reference value of 1.0, indicating that no single observation exerted excessive overall influence on the fitted model.

The residual plots provided reasonable support for the assumptions of normality and constant variance. Although several observations exhibited elevated DFFITS values, none exceeded the leverage screening threshold or had a Cook′s distance greater than 1.0. Consequently, the diagnostic results indicated that the full quadratic model was generally adequate for describing the response within the investigated experimental region, although predictions associated with influential observations should be interpreted with appropriate caution.

### 3.2. Response Surface Analysis and Extraction Mechanism

The three‐dimensional response surface plots derived from the full quadratic model (Figure 3) illustrate the combined effects of selected process variables on vanillin content. Because none of the two‐factor interaction terms was statistically significant, these plots should be interpreted as graphical representations of the simultaneous variation of two factors, whereas the remaining factors were held constant, rather than as evidence of statistically significant interaction effects.

**Figure 3:**
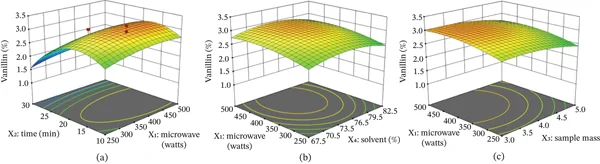
Response surface plots illustrating the combined effects of (a) microwave power and extraction time, (b) microwave power and ethanol concentration, and (c) microwave power and sample mass on vanillin content, as predicted by the full quadratic model.

Figure 3a shows the combined effects of microwave power and extraction time on vanillin content. The response surface exhibited a distinct maximum at intermediate levels of both variables, consistent with the significant negative quadratic coefficients of microwave power and extraction time in Equation (7). At low microwave power, insufficient microwave energy may limit disruption of the plant cell structure and reduce the release of vanillin into the extraction solvent. Conversely, excessive microwave power or prolonged extraction time may expose the extract to excessive thermal conditions, potentially promoting vanillin degradation and consequently reducing its recovery [25, 34]. These findings indicate that vanillin recovery was governed by a balance between enhanced microwave‐induced mass transfer and the adverse effects of excessive thermal exposure.

Figure 3b illustrates the combined effects of microwave power and ethanol concentration. The pronounced curvature along the ethanol concentration axis was consistent with the significant negative quadratic effect of ethanol concentration ($X_{4}^{2}$). Although the linear effect of ethanol concentration (*X*_4_) was positive in the full model, it was not statistically significant. Therefore, the effect of ethanol concentration should be interpreted primarily through the pronounced quadratic curvature of the response surface, which indicates decreased vanillin content as the solvent concentration deviated from the intermediate region. An ethanol concentration of approximately 75% was located within the region associated with greater vanillin recovery. This intermediate ethanol concentration may provide an appropriate balance between vanillin solubility and the dielectric properties required for effective microwave energy absorption [35]. The water fraction in the aqueous ethanol solution may also facilitate hydration and swelling of the plant cell walls, thereby improving solvent penetration and vanillin mass transfer.

Figure 3c shows the combined effects of microwave power and sample mass. The response surface indicated that vanillin recovery was maximized at an intermediate sample mass, consistent with the significant negative linear and quadratic effects of sample mass observed in the full model. At higher solid loadings, nonuniform microwave energy distribution, reduced solvent penetration into the plant matrix, and increased resistance to mass transfer may limit vanillin recovery [32]. Therefore, maintaining an appropriate sample mass relative to the fixed solvent volume was important for achieving effective microwave energy distribution and solid–liquid contact during extraction. The response surface plots confirmed the presence of pronounced curvature in the effects of the investigated variables and supported the existence of intermediate operating regions for maximizing vanillin recovery. The specific optimal conditions obtained through numerical optimization are presented in Section 3.3.

### 3.3. Monte Carlo Uncertainty Analysis, Sensitivity Analysis, and Optimal Condition Determination

Numerical optimization of the full quadratic model identified the operating conditions that maximized the predicted vanillin yield. The resulting optimal conditions were 436 W microwave power, 16.93 min extraction time, 3.45 g sample mass on a DW basis, and 75.0% ethanol, as summarized in Table 2. The Monte Carlo simulation, comprising 10,000 iterations, was performed to quantify the uncertainty associated with the model predictions under the optimized extraction conditions [36]. The distribution of the predicted vanillin yield is shown in Figure 4. The analysis yielded a mean predicted optimal yield of 31.29 mg/g DW (3.13% DW), with a Monte Carlo–derived 95% uncertainty interval of 29.41–33.16 mg/g DW (2.94%–3.32% DW). The half‐width of this interval corresponded to a relative uncertainty of approximately ±6.0% of the mean predicted value. These results indicate that the predicted response remained within a relatively narrow range after propagation of model parameter, analytical, and operational uncertainties. This uncertainty‐based evaluation complements previous MAE studies that extended process optimization toward scale‐up simulation and profitability risk analysis [37]. As illustrated in Figure 4, the red dashed line represents the mean predicted yield of 31.29 mg/g DW, whereas the shaded region represents the Monte Carlo–derived 95% uncertainty interval of 29.41–33.16 mg/g DW. The predicted response and its associated uncertainty interval are also summarized in Table 2.

**Figure 4:**
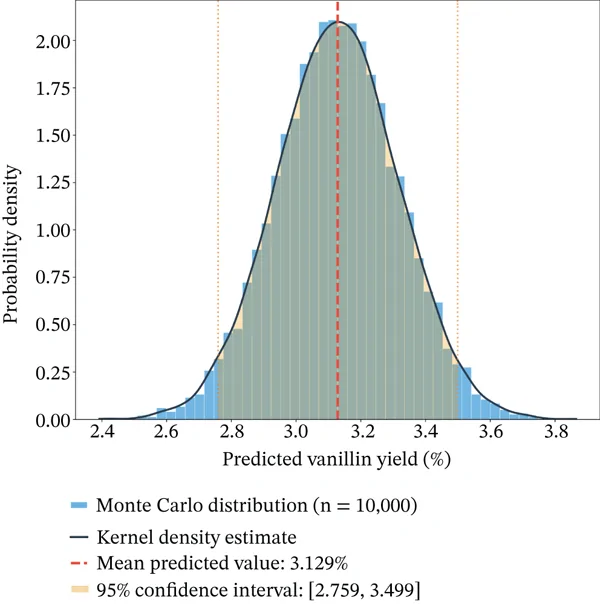
Probability distribution of the predicted vanillin yield under the optimized CMAE conditions obtained from Monte Carlo simulation (*n* = 10,000).

Sensitivity analysis of the Monte Carlo outputs using SRCs identified microwave power and ethanol concentration as the most influential process variables, as presented in Table 3. Microwave power exhibited an absolute SRC (|SRC|) of 0.45 and a relative importance of 50.0% based on the normalized squared SRC, whereas ethanol concentration exhibited an |SRC| of 0.38 and a relative importance of 35.6%. Together, these two variables represented 85.6% of the total relative importance among the four process variables included in the sensitivity analysis. Extraction time showed a smaller influence, with an |SRC| of 0.22 and a relative importance of 11.9%, whereas sample mass had the lowest influence, with an |SRC| of 0.10 and a relative importance of 2.5%.

The sensitivity ranking was broadly consistent with the ANOVA results presented in Table 4, particularly the significant quadratic effects of microwave power and ethanol concentration, which exhibited *F*‐values of 30.87 and 41.75, respectively. However, ANOVA and SRC analysis provide different information: ANOVA evaluates the statistical significance of individual model terms, whereas SRC analysis evaluates the influence of variability in the sampled process variables on the simulated response.

From a process‐control perspective, these results indicate that microwave power delivery and ethanol concentration should be carefully controlled to improve the reproducibility of vanillin recovery. The IoT‐based solid‐state relay, with a power‐control precision of ±5 W, and the solvent preparation procedure, with an ethanol concentration tolerance of ±0.5% (v/v), were therefore considered appropriate control strategies under the investigated laboratory‐scale conditions.

The Monte Carlo procedure, in which numerical optimization was repeated for each sampled full‐model realization, generated uncertainty distributions for both the optimal process variables and the corresponding predicted yield. The optimal values, their Monte Carlo–derived uncertainty intervals, and the corresponding practical operating settings are presented in Table 2. The optimized extraction time of 16.93 min was rounded to a practical setting of 17.0 min, whereas the remaining variables could be implemented at or near their numerically optimized values.

The optimal microwave power of 436 W was associated with a Monte Carlo–derived 95% uncertainty interval of 421.5–451.2 W. The corresponding intervals for extraction time, sample mass, and ethanol concentration were 16.1–17.8 min, 3.31–3.59 g DW, and 72.4%–77.6%, respectively. These intervals represent the uncertainty ranges generated by the Monte Carlo procedure and should be interpreted within the investigated experimental design space rather than as evidence of industrial‐scale process capability.

Representative HPLC chromatograms of an authentic vanillin standard at 50 *μ*g/mL and the *Vanilla planifolia* extract obtained under the optimized CMAE conditions are presented in Figure 5. The vanillin peak in the extract was identified by comparing its retention time with that of the authentic standard under identical chromatographic conditions. The chromatographic results confirmed the presence of the target vanillin peak in the extract obtained at 436 W microwave power, 16.93 min extraction time, 3.45 g sample mass on a DW basis, and 75% ethanol.

**Figure 5:**
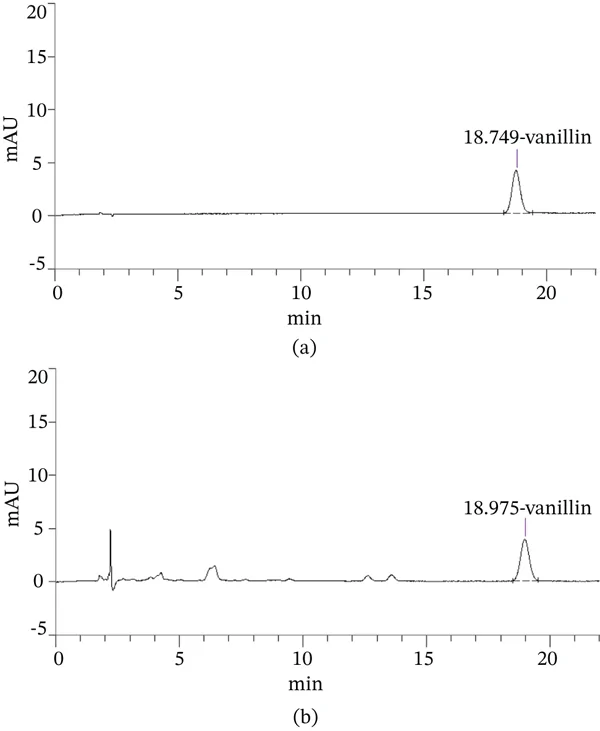
Representative HPLC chromatograms of (a) an authentic vanillin standard at 50 *μ*g/mL and (b) a *Vanilla planifolia* extract obtained under the optimized CMAE conditions of 436 W microwave power, 16.93 min extraction time, 3.45 g sample mass on a dry‐weight basis, and 75% ethanol. Detection was performed at 254 nm.

### 3.4. Validation of Optimized Conditions and Energy Equivalence

The optimized operating region was validated using two approximately energy‐equivalent power–time combinations: 380 W for 19.44 min and 500 W for 14.78 min. These conditions were selected to provide nominal power–time products comparable to that of the numerically optimized condition of 436 W for 16.93 min. The corresponding power–time products were 7387, 7390, and 7381 W·min, respectively. Because the same cyclic duty cycle of 25‐s ON and 5‐s OFF was applied to all conditions, their calculated active microwave energy inputs were also approximately equivalent [38, 39].

The experimental validation results are presented in Table 5. The mean vanillin yields were 30.43 ± 0.24 mg/g DW at 380 W for 19.44 min and 30.98 ± 0.32 mg/g DW at 500 W for 14.78 min. These values deviated by 2.75% and 0.99%, respectively, from the optimized model prediction of 31.29 mg/g DW. Both experimental values were within the Monte Carlo–derived 95% uncertainty interval of 29.41–33.16 mg/g DW reported in Section 3.3. The close agreement between the experimental values and the optimized model prediction supports the predictive adequacy of the full quadratic model within the investigated experimental region and demonstrates that comparable vanillin yields could be obtained using different, approximately energy‐equivalent power–time settings. Nevertheless, these results should not be interpreted as demonstrating that total energy input alone determines vanillin yield, because microwave power and extraction time remained independent variables in the fitted quadratic model.

**Table 5:** Validation of the optimized CMAE conditions under approximately energy‐equivalent power–time settings.

Condition (W/min)	Vanillin yield (mg/g DW)	*M* *e* *a* *n* ± *S* *D*	TPC (mg GAE/g DW)	*M* *e* *a* *n* ± *S* *D*
380 W/19.44 min	30.43	±0.24	27.62	±0.17
500 W/14.78 min	30.98	±0.32	32.10	±0.02
Model prediction	31.29	—	—	—

The TPC values were 27.62 ± 0.17 mg GAE/g DW at 380 W for 19.44 min and 32.10 ± 0.02 mg GAE/g DW at 500 W for 14.78 min. Although the two settings provided similar vanillin yields, the observed difference in TPC indicates that approximately equivalent microwave energy inputs did not necessarily produce identical coextraction behavior. The higher power, shorter time condition resulted in a higher TPC, suggesting that the specific power–time combination may influence the recovery of phenolic compounds even when the nominal microwave energy input is comparable. These findings are consistent with previous reports indicating that MAE conditions can affect both target‐compound recovery and the extraction of other phenolic constituents [11, 14].

### 3.5. Calculated Energy Performance and Sustainability Implications

Under the optimized CMAE conditions (436 W, 16.93 min, and a duty cycle of 25‐s ON/5‐s OFF), the cumulative microwave ON‐time was calculated as follows:

(8) TON=16.9314.11×25255+= min.

The corresponding nominal microwave energy delivered during the ON periods was as follows:

(9) Ecyclic=43614.116152369.1 W× min= W min= kJ.

For the calculated continuous‐operation baseline at the same nominal microwave power and total extraction time, the calculated microwave energy input was as follows:

(10) Econtinuous=43616.937381.5442.9 W× min= W min= kJ.

The calculated reduction in nominal microwave energy was therefore as follows:

(11) Energy reduction=442.9369.1−442.9×10016.7=%.

Thus, the cyclic duty cycle reduced the calculated active irradiation time and nominal microwave energy input by 16.7% relative to the calculated uninterrupted operation baseline at the same nominal power and total extraction time. This comparison represents the energy associated with the programmed microwave power and operating duration and does not include magnetron efficiency, control system electricity, cooling requirements, or other auxiliary electrical loads. The mass of vanillin recovered per batch was calculated from the model‐predicted vanillin yield and the dry mass of vanilla beans:

(12) mvanillin=31.293.45107.950.10795 mg/g  DW× g DW= mg= g.

Accordingly, the SEC for CMAE was as follows:

(13) SECcyclic=369.1 kJ0.10795 g=3419.2kJgvanillin.

For the calculated continuous‐operation baseline, assuming the same vanillin recovery of 0.10795 g per batch, the corresponding SEC was as follows:

(14) SECcontinuous=442.9 kJ0.10795 g=4102.8 kJg vanillin.

As summarized in Table 6, cyclic operation resulted in a calculated 16.7% reduction in nominal microwave energy input relative to the continuous‐operation baseline. Under the assumption of equivalent vanillin recovery, the calculated SEC was reduced by the same proportion. Because the continuous‐operation yield was assumed rather than experimentally determined, the SEC comparison represents a theoretical equal‐recovery scenario. The calculated energy and SEC values for both operating modes are compared in Table 6.

**Table 6:** Calculated nominal microwave energy and specific energy consumption of CMAE and the continuous‐operation baseline.

Parameter	CMAE (cyclic)	Continuous operation (calculated baseline)	Reduction (%)
Applied microwave power (W)	436	436	—
Total extraction time (min)	16.93	16.93	—
Duty factor	0.833	1.000	16.7
Active irradiation time (min)	14.11	16.93	16.7
Calculated nominal microwave energy input (kJ per batch)	369.1	442.9	16.7
Assumed vanillin recovery (g per batch)	0.10795	0.10795	—
Calculated SEC (kJ g^−1^ vanillin)	3419.2	4102.8	16.7

As shown in Table 6, the calculated SEC decreased from 4102.8 to 3419.2 kJ/g vanillin. The identical percentage reductions in nominal microwave energy input and SEC resulted directly from the assumption that both operating modes achieved the same vanillin recovery. During the OFF periods, microwave irradiation was interrupted, whereas residual thermal energy in the extraction system may have continued to support heat and mass transfer [40, 41]. This mechanism may allow extraction to continue without uninterrupted microwave irradiation. However, because the continuous‐operation baseline and its vanillin recovery were calculated rather than experimentally measured, the results indicate a potential reduction in nominal microwave energy demand rather than a confirmed improvement in extraction efficiency. Further validation using experimentally measured vanillin yields and wall‐plug electrical energy consumption for both cyclic and continuous operating modes is required.

### 3.6. Industrial Implications and Scalability

The IoT‐integrated CMAE system demonstrates potential for future scale‐up and smart process monitoring but should not yet be regarded as industrially validated. Its modular configuration may support future parallel reactor configurations; however, larger scale validation and process modeling are required to address microwave‐field uniformity and thermal gradients in larger extraction vessels and microwave cavities [42]. The ESP32–Node‐RED platform could facilitate centralized monitoring and automated data logging and digital traceability, consistent with Industry 4.0 processing concepts [14, 43]. Similar vanillin yields obtained using two approximately energy‐equivalent power–time combinations also indicate a degree of operational flexibility within the investigated laboratory‐scale design space. Moreover, cyclic operation produced a calculated 16.7% reduction in nominal microwave energy input and SEC relative to the calculated continuous‐operation baseline under the equal‐recovery assumption. This calculated advantage should be interpreted as a preliminary indication of potential energy savings rather than as confirmation of improved industrial energy efficiency. Pilot‐scale testing, measured electrical energy consumption, experimental comparison with continuous microwave operation, and comprehensive technoeconomic assessment are therefore required before industrial implementation.

### 3.7. Limitations and Future Research Directions

This study was limited to a laboratory‐scale system using 2–6 g DW of vanilla beans per 100 mL of solvent and samples from a single geographic origin. Pilot‐scale validation is required to assess microwave‐field uniformity, thermal gradients, extraction performance, process reproducibility, and measured wall‐plug energy consumption at larger capacities [44]. In addition, the continuous‐operation baseline and its SEC were calculated under an equal‐recovery assumption rather than determined experimentally. Future studies should therefore compare cyclic and continuous microwave operating modes using experimentally measured vanillin yields and wall‐plug electrical energy consumption, while accounting for magnetron efficiency, control system electricity, cooling requirements, and other auxiliary energy demands. Further research should also evaluate vanilla beans from multiple geographic origins [45] and conduct life cycle and technoeconomic assessments [46]. Future system development could incorporate additional process sensors and adaptive control algorithms to improve real‐time process monitoring and enable data‐driven adjustment of operating conditions. The applicability of the CMAE platform to other high‐value botanical materials should also be investigated.

## 4. Conclusion

This study developed an IoT‐integrated CMAE system for vanillin recovery from *Vanilla planifolia*. The system enabled real‐time temperature monitoring and reproducible programmable cyclic operation. RSM identified optimal conditions of 436 W, 16.93 min, 3.45 g DW, and 75% ethanol, with a predicted vanillin content of 3.13% DW, equivalent to 31.29 mg/g DW. Monte Carlo analysis yielded a Monte Carlo–derived 95% uncertainty interval of 29.41–33.16 mg/g DW, whereas experimental validation showed deviations of 2.75% and 0.99% from the model prediction. Assuming equivalent vanillin recovery, cyclic operation reduced the calculated nominal microwave energy input and SEC by 16.7% relative to a calculated continuous‐operation baseline. Overall, the CMAE system demonstrates potential for reducing nominal microwave energy demand while supporting real‐time process monitoring and flexible cyclic operation at the laboratory scale. However, direct experimental comparison with continuous microwave operation, measurement of wall‐plug electrical energy consumption, and pilot‐scale validation remain necessary to confirm its energy performance and industrial scalability.

## Author Contributions

**Narongrit Tipcompor:** conceptualization, methodology, investigation, and writing—review and editing; **Thanapon Saengsuwan:** methodology, data curation, formal analysis, supervision, and writing—review and editing; **Narathip Sujinda:** methodology, formal analysis, and writing—review and editing; **Sujitra Funsueb:** methodology; **Watcharaporn Thaharn:** conceptualization, methodology, investigation, resources, funding acquisition, supervision, and writing—review and editing.

## Funding

This work was supported by the National Science, Research and Innovation Fund (NSRF) through Chiang Rai Rajabhat University (FF68‐1‐001).

## Conflicts of Interest

The authors declare no conflicts of interest.

## Supporting Information

Additional supporting information can be found online in the Supporting Information section.

## Supporting information


**Supporting Information 1** Figure S1: Calibration curve for vanillin quantification obtained by HPLC‐PDA detection at 254 nm over the concentration range of 25–200 mg/L.

**Supporting Information 2** Table S1: Vanillin content (%) obtained under the optimal conditions predicted by the CCD model using cyclic microwave‐assisted extraction (CMAE).

**Supporting Information 3** Figure S2: (a) Normal probability plot of internally studentized residuals. (b) Plot of residuals‐versus‐predicted values.

**Supporting Information 4** Table S2: Influence diagnostics for the quadratic model.

## Data Availability

The data that support the findings of this study are available from the corresponding author upon reasonable request.
