# Supplementary material for: Process Optimization and Uncertainty Analysis of IoT‐Integrated Cyclic Microwave‐Assisted Extraction (CMAE) for Vanillin Recovery
Source: Int J Food Sci. 2026 Sep 28;2026:4384246. doi: 10.1155/ijfo/4384246 (PMC13620407; doi:10.1155/ijfo/4384246)
Supplement: Supplementary file 1 [file IJFO-2026-4384246-s001.docx]

**TABLE S2** Influence diagnostics for quadratic model.

| **Run Order** | **Actual Value** | **Predicted Value** | **Residual** | **Leverage** | **Internally Studentized Residuals** | **Externally Studentized Residuals** | **Cook's Distance** | **Influence on Fitted Value DFFITS** | **Standard Order** |
| --- | --- | --- | --- | --- | --- | --- | --- | --- | --- |
| 1 | 2.32 | 2.30 | 0.0273 | 0.589 | 0.228 | 0.221 | 0.005 | 0.264 | 19 |
| 2 | 2.38 | 2.34 | 0.0412 | 0.589 | 0.343 | 0.334 | 0.011 | 0.399 | 12 |
| 3 | 1.62 | 1.75 | -0.1252 | 0.589 | -1.044 | -1.047 | 0.104 | -1.252 | 22 |
| 4 | 2.26 | 2.02 | 0.2365 | 0.589 | 1.972 | 2.195 | 0.371 | 2.625⁽¹⁾ | 10 |
| 5 | 2.52 | 2.45 | 0.0703 | 0.589 | 0.587 | 0.574 | 0.033 | 0.687 | 28 |
| 6 | 2.56 | 2.62 | -0.0523 | 0.589 | -0.436 | -0.425 | 0.018 | -0.508 | 13 |
| 7 | 1.75 | 1.92 | -0.1733 | 0.589 | -1.445 | -1.501 | 0.199 | -1.795 | 20 |
| 8 | 2.34 | 2.31 | 0.0230 | 0.589 | 0.192 | 0.186 | 0.004 | 0.222 | 29 |
| 9 | 2.39 | 2.50 | -0.1091 | 0.569 | -0.890 | -0.883 | 0.070 | -1.016 | 3 |
| 10 | 2.49 | 2.33 | 0.1631 | 0.569 | 1.330 | 1.365 | 0.156 | 1.569 | 16 |
| 11 | 2.28 | 2.25 | 0.0301 | 0.569 | 0.246 | 0.238 | 0.005 | 0.274 | 31 |
| 12 | 2.16 | 2.31 | -0.1497 | 0.569 | -1.220 | -1.241 | 0.131 | -1.426 | 25 |
| 13 | 2.47 | 2.74 | -0.2625 | 0.569 | -2.140 | -2.452 | 0.403 | -2.819⁽¹⁾ | 4 |
| 14 | 2.74 | 2.69 | 0.0419 | 0.569 | 0.341 | 0.332 | 0.010 | 0.381 | 14 |
| 15 | 2.36 | 2.50 | -0.1364 | 0.569 | -1.112 | -1.121 | 0.109 | -1.288 | 30 |
| 16 | 2.64 | 2.69 | -0.0466 | 0.569 | -0.380 | -0.369 | 0.013 | -0.425 | 21 |
| 17 | 2.03 | 2.13 | -0.0931 | 0.510 | -0.712 | -0.701 | 0.035 | -0.715 | 5 |
| 18 | 2.53 | 2.41 | 0.1196 | 0.779 | 1.361 | 1.401 | 0.435 | 2.629⁽¹⁾ | 9 |
| 19 | 1.96 | 2.07 | -0.1098 | 0.577 | -0.903 | -0.898 | 0.074 | -1.049 | 7 |
| *20* | *2.82* | *2.61* | *0.2152* | *0.577* | *1.770* | *1.912* | *0.285* | *2.233⁽¹⁾* | *24* |
| 21 | 2.79 | 2.80 | -0.0127 | 0.577 | -0.104 | -0.101 | 0.001 | -0.118 | 2 |
| 22 | 2.38 | 2.26 | 0.1181 | 0.577 | 0.971 | 0.970 | 0.086 | 1.132 | 15 |
| 23 | 2.30 | 2.02 | 0.2866 | 0.577 | 2.358 | 2.827 | 0.506 | 3.302⁽¹⁾ | 1 |
| 24 | 2.05 | 2.24 | -0.1813 | 0.577 | -1.491 | -1.556 | 0.202 | -1.817 | 11 |
| 25 | 2.97 | 3.04 | -0.0661 | 0.141 | -0.382 | -0.371 | 0.002 | -0.150 | 8 |
| 26 | 3.09 | 3.04 | 0.0555 | 0.141 | 0.321 | 0.311 | 0.001 | 0.126 | 18 |
| 27 | 2.99 | 3.04 | -0.0464 | 0.141 | -0.268 | -0.260 | 0.001 | -0.105 | 6 |
| 28 | 3.02 | 3.04 | -0.0166 | 0.141 | -0.096 | -0.093 | 0.000 | -0.038 | 23 |
| *29* | *3.29* | *3.04* | *0.2592* | *0.141* | *1.496* | *1.562* | *0.024* | *0.633* | *17* |
| 30 | 2.99 | 3.04 | -0.0437 | 0.141 | -0.252 | -0.245 | 0.001 | -0.099 | 27 |
| 31 | 2.97 | 3.04 | -0.0630 | 0.141 | -0.363 | -0.353 | 0.001 | -0.143 | 26 |

⁽¹⁾ Exceeds the DFFITS screening threshold of $\pm1.936$, calculated as $2\sqrt{p/\left( n-p \right)}$, where $p=15$ and $n=31$.
