# Supplementary material for: Process Optimization and Uncertainty Analysis of IoT‐Integrated Cyclic Microwave‐Assisted Extraction (CMAE) for Vanillin Recovery
Source: Int J Food Sci. 2026 Sep 28;2026:4384246. doi: 10.1155/ijfo/4384246 (PMC13620407; doi:10.1155/ijfo/4384246)
Supplement: Supplementary file 2 [file IJFO-2026-4384246-s002.docx]

**FIGURE S2** (a) Normal probability plot of internally studentized residuals; (b) Plot of residuals versus predicted values.

(a) (b)
