# Supplementary material for: Process Optimization and Uncertainty Analysis of IoT‐Integrated Cyclic Microwave‐Assisted Extraction (CMAE) for Vanillin Recovery
Source: Int J Food Sci. 2026 Sep 28;2026:4384246. doi: 10.1155/ijfo/4384246 (PMC13620407; doi:10.1155/ijfo/4384246)
Supplement: Supplementary file 3 [file IJFO-2026-4384246-s003.docx]

**TABLE S1** Vanillin content (%) obtained under the optimal conditions predicted by the CCD model using cyclic microwave-assisted extraction (CMAE).

| Treatment | Power (W) | Time (min) | Mass  (g DW) | Ethanol (%) | Factor 1 Power (W) | Factor 2  Time (min) | Factor 3  Mass  (g DW) | Factor 4  Ethanol (%) | Vanillin content (%) | | | |
| --- | --- | --- | --- | --- | --- | --- | --- | --- | --- | --- | --- | --- |
|  |  |  |  |  |  |  |  |  | 1 | 2 | 3 | average |
| 1 | -1 | -1 | -1 | -1 | 250 | 10 | 3 | 67.5 | 2.3916 | 2.3002 | 2.2811 | 2.3243 |
| 2 | -1 | -1 | -1 | 1 | 250 | 10 | 3 | 82.5 | 2.3991 | 2.3556 | 2.3753 | 2.3767 |
| 3 | -1 | -1 | 1 | -1 | 250 | 10 | 5 | 67.5 | 1.6176 | 1.6233 | 1.632 | 1.6241 |
| 4 | -1 | -1 | 1 | 1 | 250 | 10 | 5 | 82.5 | 2.2291 | 2.2904 | 2.2492 | 2.2562 |
| 5 | -1 | 1 | -1 | -1 | 250 | 20 | 3 | 67.5 | 2.4948 | 2.5797 | 2.4988 | 2.5244 |
| 6 | -1 | 1 | -1 | 1 | 250 | 20 | 3 | 82.5 | 2.5778 | 2.5562 | 2.5581 | 2.5640 |
| 7 | -1 | 1 | 1 | -1 | 250 | 20 | 5 | 67.5 | 1.7559 | 1.7276 | 1.7586 | 1.7474 |
| 8 | -1 | 1 | 1 | 1 | 250 | 20 | 5 | 82.5 | 2.3153 | 2.3487 | 2.3494 | 2.3378 |
| 9 | 1 | -1 | -1 | -1 | 500 | 10 | 3 | 67.5 | 2.4123 | 2.3677 | 2.3867 | 2.3889 |
| 10 | 1 | -1 | -1 | 1 | 500 | 10 | 3 | 82.5 | 2.5015 | 2.4428 | 2.5396 | 2.4946 |
| 11 | 1 | -1 | 1 | -1 | 500 | 10 | 5 | 67.5 | 2.2546 | 2.2801 | 2.2945 | 2.2764 |
| 12 | 1 | -1 | 1 | 1 | 500 | 10 | 5 | 82.5 | 2.1686 | 2.157 | 2.1604 | 2.1620 |
| 13 | 1 | 1 | -1 | -1 | 500 | 20 | 3 | 67.5 | 2.5226 | 2.4511 | 2.45 | 2.4746 |
| 14 | 1 | 1 | -1 | 1 | 500 | 20 | 3 | 82.5 | 2.7667 | 2.8026 | 2.639 | 2.7361 |
| 15 | 1 | 1 | 1 | -1 | 500 | 20 | 5 | 67.5 | 2.3567 | 2.3322 | 2.4005 | 2.3631 |
| 16 | 1 | 1 | 1 | 1 | 500 | 20 | 5 | 82.5 | 2.6886 | 2.5573 | 2.6804 | 2.6421 |
| 17 | -2 | 0 | 0 | 0 | 120 | 15 | 4 | 75 | 2.0159 | 2.0239 | 2.064 | 2.0346 |
| 18 | 2 | 0 | 0 | 0 | 700 | 15 | 4 | 75 | 2.5383 | 2.5309 | 2.5328 | 2.5340 |
| 19 | 0 | -2 | 0 | 0 | 380 | 5 | 4 | 75 | 1.9389 | 1.9739 | 1.9738 | 1.9622 |
| 20 | 0 | 2 | 0 | 0 | 380 | 25 | 4 | 75 | 2.7989 | 2.8795 | 2.7952 | 2.8245 |
| 21 | 0 | 0 | -2 | 0 | 380 | 15 | 2 | 75 | 2.7982 | 2.8361 | 2.7411 | 2.7918 |
| 22 | 0 | 0 | 2 | 0 | 380 | 15 | 6 | 75 | 2.3089 | 2.399 | 2.4356 | 2.3812 |
| 23 | 0 | 0 | 0 | -2 | 380 | 15 | 4 | 60 | 2.3449 | 2.2342 | 2.33 | 2.3030 |
| 24 | 0 | 0 | 0 | 2 | 380 | 15 | 4 | 90 | 2.0622 | 2.0946 | 2.0069 | 2.0546 |
| 25 | 0 | 0 | 0 | 0 | 380 | 15 | 4 | 75 | 3.0448 | 2.9371 | 2.9261 | 2.9693 |

| Treatment | Power (W) | Time (min) | Mass (g) | Ethanol (%) | Factor 1 Power (W) | Factor 2  Time (min) | Factor 3  Mass (g) | Factor 4  Ethanol (%) | Vanillin rate in % mass | | | |
| --- | --- | --- | --- | --- | --- | --- | --- | --- | --- | --- | --- | --- |
|  |  |  |  |  |  |  |  |  | 1 | 2 | 3 | average |
| 26 | 0 | 0 | 0 | 0 | 380 | 15 | 4 | 75 | 3.0217 | 2.9926 | 3.2587 | 3.0910 |
| 27 | 0 | 0 | 0 | 0 | 380 | 15 | 4 | 75 | 2.9805 | 3.0269 | 2.9597 | 2.9890 |
| 28 | 0 | 0 | 0 | 0 | 380 | 15 | 4 | 75 | 2.9744 | 3.1574 | 2.9248 | 3.0189 |
| 29 | 0 | 0 | 0 | 0 | 380 | 15 | 4 | 75 | 3.283 | 3.3424 | 3.2587 | 3.2947 |
| 30 | 0 | 0 | 0 | 0 | 380 | 15 | 4 | 75 | 2.9371 | 3.1484 | 2.8898 | 2.9918 |
| 31 | 0 | 0 | 0 | 0 | 380 | 15 | 4 | 75 | 2.9786 | 3.0599 | 2.879 | 2.9725 |
