# Supplementary material for: Process Optimization and Uncertainty Analysis of IoT‐Integrated Cyclic Microwave‐Assisted Extraction (CMAE) for Vanillin Recovery
Source: Int J Food Sci. 2026 Sep 28;2026:4384246. doi: 10.1155/ijfo/4384246 (PMC13620407; doi:10.1155/ijfo/4384246)
Supplement: Supplementary file 4 [file IJFO-2026-4384246-s004.docx]

**FIGURE S1** Calibration curve for vanillin quantification obtained by HPLC–PDA detection at 254 nm over the concentration range of 25–200 mg/L.

The extraction experiments were designed using a Central Composite Design (CCD) with four independent variables: microwave power (X₁, 120–700 W), extraction time (X₂, 5–25 min), sample mass (X₃, 2–6 g DW), and ethanol concentration (X₄, 60–90% v/v).

Vanillin content in the extracts was quantified using high-performance liquid chromatography equipped with a photodiode array detector (HPLC–PDA) operating at 254 nm. Chromatographic separation was carried out on a C18 reversed-phase column (4.6 × 100 mm, 2.7 µm) using an isocratic mobile phase consisting of methanol and acidified water (15:85, v/v), with the aqueous phase containing 0.1% (v/v) acetic acid, at a flow rate of 0.5 mL min⁻¹. The injection volume was 1 µL, and the column temperature was maintained at 30 °C.

Quantification was performed using an external standard calibration method with vanillin standard solutions ranging from 25 to 200 mg/L. The calibration curve (Figure S1) exhibited excellent linearity and was described by the following regression equation:

$$y=2.17256x-18.3842 \left( R^{2}=0.99995 \right)$$

where *y* represents the chromatographic peak area (mAU·s) and *x* represents the vanillin concentration (mg/L). The calibration model exhibited an excellent coefficient of determination (R² = 0.99995) and a relative standard error (RSE = 1.19%), confirming the accuracy, linearity, and precision of the analytical method over the investigated concentration range. All measurements were performed in triplicate, and the results are expressed as the mean ± standard deviation.
